# Two Simultaneous Leak Diagnosis in Pipelines Based on Input–Output Numerical Differentiation

**DOI:** 10.3390/s21238035

**Published:** 2021-12-01

**Authors:** Adrián Navarro-Díaz, Jorge-Alejandro Delgado-Aguiñaga, Ofelia Begovich, Gildas Besançon

**Affiliations:** 1 Tecnologico de Monterrey, School of Engineering and Sciences, Av. General Ramón Corona 2514, Zapopan C.P. 45138, Mexico; adrian.navarro@tec.mx; 2 Centro de Investigación, Innovación y Desarrollo Tecnológico CIIDETEC-UVM, Universidad del Valle de México, Periférico Sur Manuel Gómez Morín 8077, Tlaquepaque C.P. 45601, Mexico; 3 CINVESTAV Guadalajara, Av. del Bosque 1145, Col. El Bajío, Zapopan C.P. 45019, Mexico; ofelia.begovich@cinvestav.mx; 4 GIPSA-Lab, Institute of Engineering, Université Grenoble Alpes, CNRS, Grenoble IPN, 38000 Grenoble, France; gildas.besancon@gipsa-lab.grenoble-inp.fr

**Keywords:** fault diagnosis, pipelines, multiple leaks, numerical differentiation, experimental results

## Abstract

This paper addresses the two simultaneous leak diagnosis problem in pipelines based on a state vector reconstruction as a strategy to improve water shortages in large cities by only considering the availability of the flow rate and pressure head measurements at both ends of the pipeline. The proposed algorithm considers the parameters of both leaks as new state variables with constant dynamics, which results in an extended state representation. By applying a suitable persistent input, an invertible mapping in *x* can be obtained as a function of the input and output, including their time derivatives of the third-order. The state vector can then be reconstructed by means of an algebraic-like observer through the computation of time derivatives using a Numerical Differentiation with Annihilatorsconsidering its inherent noise rejection properties. Experimental results showed that leak parameters were reconstructed with accuracy using a test bed plant built at Cinvestav Guadalajara.

## 1. Introduction

In recent decades, climate change and the overuse of natural water resources have caused water scarcity in big cities. Furthermore, water distribution systems operators (WDSOs) are facing major water losses as high as 65% due to pipeline leaks caused by lack of maintenance, illegal intrusion, or accidents.

**Figure 1:**
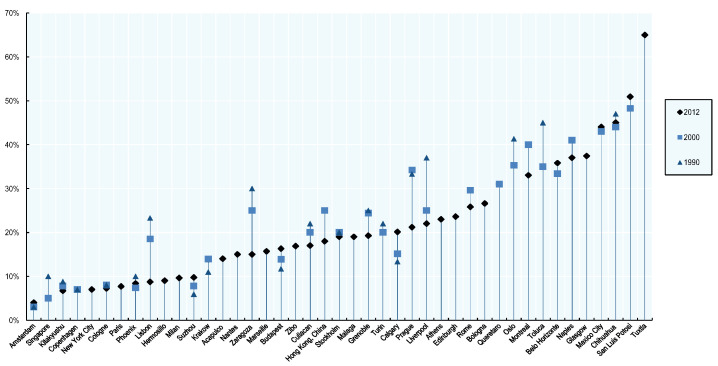
Proportion of water loss in surveyed cities (leakage rate).

According to a study performed by the Organisation for Economic Co-operation (OECD), entitled Water Governance in Cities [1], aging water networks have a negative impact in terms of efficiency. One of the consequences is water loss from pipeline leaks. On average, water loss in the surveyed cities (in the referred report) was 21% in 2016. However, for Mexican cities, water loss was more than 40% (Chihuahua, Mexico City, San Luis Potosi) or even up to 65% (Tuxtla), see Figure 1.

On the other hand, to satisfy the current demand, government policies are focused on bringing more water from far away places instead of solving water losses due to leaks. This means that the amount of water lost is currently considered in the water budget. Interestingly, the amount of water needed to meet the demand in deficit is very similar to what is lost through leaks. In other words, it could be possible to satisfy the current water demand by minimizing the water losses due to leaks without the need for bringing more water from far away places.

The implementation of leak detection and isolation (*LDI*) systems has demonstrated a reduction in water losses in pipelines. *LDI* systems are algorithms that perform the following tasks: *detection* and *localization* of one or several leaks in a pipeline system. Thus, once a leak is identified, the repair technicians can fix the leak and avoid the water loss. Many works that address the *LDI* problem for one leak have been reported [2,3,4,5,6]. For instance, in [2], a leak isolation methodology using a fitting loss coefficient calibration is presented on the basis of two stages: in the first stage, the equivalent straight length (*ESL*) is fixed by a model-based observer designed as an extended Kalman filter (*EKF*); in the second stage, an algebraic observer is started with the *ESL* value fixed by the previous observer. Finally, the estimated leak position is recovered in original coordinates since the observer deals with *ESL* coordinates. Authors in [3] presented a methodology for leak detection and isolation in pipelines based on data fusion using two approaches: a steady-state estimation and an *EKF*. Authors concluded that the solution of the *LDI* problem improves significantly when a steady-state estimation is incorporated to the estimation provided by the *EKF*. In other words, the solution provided by the *EKF* is less accurate by itself. In [4], authors propose a bank of observers together with a Genetic Algorithm (*GA*), which is exploited to minimize the integration of the square observation error. The minimum integral observation error is reached in the observer where the estimated leak parameters match the real values. Experimental results evidence an accurate leak position estimation in a test bed pilot plant. In [5], a combined artificial neural network (*ANN*) for leak diagnosis in pipelines is presented. The *ANN* scheme estimates the location and friction factor based on measurement data. An average error of 0.629% was obtained for leak location in the experiments. More recently, in [6], a new approach for solving the *LDI* problem in pipelines is introduced on the basis of a Kalman filter for linear parameter varying (*LPV*) systems. The off-line computation of the filter gain allows the computational effort to be reduced and the authors claim that the *LPV* design outperforms the classical *EKF* design in terms of parameter-estimation accuracy.

On the other hand, the multi-leak case study has also been considered from two different perspectives: (i) for sequential leaks (non-concurrent case) [7,8,9] and (ii) for the simultaneous leak problem (concurrent case) [10].

In [7], a model adaptation strategy is proposed to isolate non-concurrent multiple leaks based on extended Kalman filters. Experimental results show the potential of this approach by allowing to monitor each new leak, no matter where it appears. Following this direction, a scheme is proposed in [8] for detecting and locating multiple sequential leaks based on a combination of an adaptive observer to identify the hydraulic gradient in real time and a leak location observer to estimate the leak position and its outflow. Experimental results of a pilot pipeline showed a satisfactory estimation in spite of operational changes and leaks. More recently, in [9], a pressure distribution analysis is proposed to diagnose the location of leaks via an experimental study and computational fluid dynamics (*CFD*) simulation. Multiple flow rate testing is conducted to detect the locations of two leaks.

In the same way, a more complex case of the multi-leak problem is when two or more leaks occur at the same time, also known as the concurrent case. In [10], the orthogonal collocation method (*OCM*) is used to obtain an approximate solution of the water hammer equations (*WHE*). An estimator is then designed based on the spatially-discretized model to detect multiple leaks by identifying their positions and leak coefficients by applying a persistent input [11]. The results are presented via simulation.

Regarding the one leak problem, state observer-based techniques have been proposed and successfully evaluated since the observer convergence is guaranteed, even by applying constant inputs. This is because the structure of underlying state-space representation fulfills a uniform observability condition (which is independent of the input) [12]. Conversely, when two or more leaks occur simultaneously, such an observability condition is no longer satisfied and the observability depends on the input. Particularly in steady state, the output obtained by two or more leaks is equivalent to the one obtained by a single *virtual* leak, which is known as indistinguishability [11].

### 1.1. Problem Statement

- In a real pipeline system, several leaks can occur and usually they are not fixed as they appear; this means that the leak problem becomes a challenging multi-leak problem known as the simultaneous leak case. In addition, this situation worsens when water management companies frequently lack flow rate and pressure head records of the leak events.
- Therefore, a methodology to address the simultaneous leak case is proposed on the basis of an input–output numerical differentiation-based strategy by applying a persistent input in the sense of [11].

### 1.2. Methods

- By considering a state-space representation of a pipeline with two leaks in which the leak parameters are considered as new state variables with constant dynamics, the extended state can be reconstructed via its expression in terms of input, output, and the corresponding time derivatives.
- A persistent input is experimentally generated via a frequency variation of the pump driver that produces a sine-like pressure signal. This persistent input allows the parameters of each leak to be reconstructed. This approach could be extended to a more general case of simultaneous leaks if the applied input is regularly persistent, such that the observability condition is guaranteed [11]. However, this approach could also be limited to physical constraints since a persistent input might cause additional leaks due to the flow transient effect that it produces.

Hereinafter, the paper is organized as follows: In Section 2, a mathematical model is derived from the well-known water hammer equations and the two simultaneous leak problem is stated. State vector reconstruction based on injection of input–output time derivatives is presented in Section 3. Experimental results are presented in Section 4 by using databases from a pilot plant built at Cinvestav Guadalajara. Finally, several conclusions and future perspectives are given in Section 5.

## 2. Preliminaries

### 2.1. Pipeline Mathematical Model

#### 2.1.1. Governing Equations

The transient fluid through a pipeline can be described by conservation mass and momentum equations known as water hammer equations, which are a couple of quasi-linear hyperbolic partial differential equations (*PDE*). Generally, *PDE* are derived considering the following assumptions: the fluid is slightly compressible, the duct wall is slightly deformable, and the convective velocity changes are negligible. The cross section and the fluid density are assumed to be constant [13]:

*Momentum Equation*

(1) $$\frac{\partial Q(z,t)}{\partial t}+gA\frac{\partial H(z,t)}{\partial z}+\mu Q\left(z,t\right)\left|Q(z,t)\right|=0$$

*Continuity Equation*

(2) $$\frac{\partial H(z,t)}{\partial t}+\frac{b^{2}}{gA}\frac{\partial Q(z,t)}{\partial z}=0$$

Here, *Q* stands for the flow rate [m${}^{3}$/s]; *H* is the pressure head [m]; *z* is the length coordinate [m]; *t* is the time coordinate [s]; *g* is the gravity acceleration [m/s${}^{2}$]; *A* is the cross-section area [m${}^{2}$]; *b* is the pressure wave speed in the fluid [m/s]; μ=τ/2ϕA, where ϕ is the inner diameter [m] and τ is the friction factor. The dynamics in Equations (1) and (2) are fully defined by related pairs of initial and boundary conditions.

#### 2.1.2. Finite Difference Approximation

**Figure 2:**
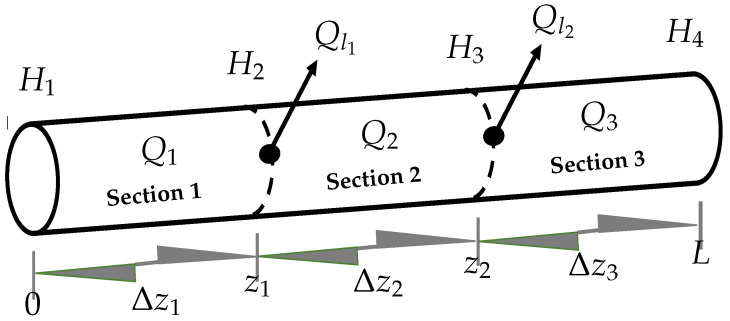
Discretization of the pipeline with two arbitrarily located leaks.

For the purpose of obtaining a finite dimensional model from (1) and (2), the partial differential equations are discretized with respect to the spatial variable *z*, as in [14,15], by using the following approximations:

(3) $$\begin{array}{c} \mathrm{Section} \end{array}i:\left\{\begin{array}{c} \frac{\partial H(z_{i},t)}{\partial z}⋍\frac{H_{i+1}-H_{i}}{\Delta z_{i}}\;\forall i=1,\cdots ,n\\ \frac{\partial Q(z_{i-1},t)}{\partial z}⋍\frac{Q_{i}-Q_{i-1}}{\Delta z_{i-1}}\;\forall i=2,\cdots ,n \end{array}\right.$$

where index *i* stands for the variable discretized in (1) and (2) at section *i*. To solve the *LDI* problem for two simultaneous leaks, Equations (1) and (2) admit a simple spatial discretization as shown in Figure 2, where sections are defined according to the two leakage positions:

Here, $Q_{l_{1,2}}$ represent the leaking flows that can be modeled as:

(4) $$Q_{l_{1,2}}=\lambda_{1,2}\sqrt{H_{2,3}}$$

where, $\lambda_{1,2}$ is a constant that depends on the orifice size and the discharge coefficient, and $H_{2,3}$ is the head pressure at the leak point.

Thus, assuming a lumped-parameter model for the flow equations and considering that the pressure head and flow rate measurements are available at both ends of the pipeline via sensors, a low order dynamical representation of the system with two leaks can be written using approximation (3) in Equations (1) and (2), as follows:

(5) $$\left[\begin{array}{c} {\dot{Q}}_{1}\\ {\dot{H}}_{2}\\ {\dot{Q}}_{2}\\ {\dot{H}}_{3}\\ {\dot{Q}}_{3} \end{array}\right]=\left[\begin{array}{c} \frac{-gA}{\Delta z_{1}}\left(H_{2}-H_{1}\right)-\frac{\tau }{2\phi A}Q_{1}\left|Q_{1}\right|\\ \frac{-b_{2}^{2}}{gA\Delta z_{1}}\left(Q_{2}-Q_{1}+\lambda_{1}\sqrt{H_{2}}\right)\\ \frac{-gA}{\Delta z_{2}}\left(H_{3}-H_{2}\right)-\frac{\tau }{2\phi A}Q_{2}\left|Q_{2}\right|\\ \frac{-b_{2}^{2}}{gA\Delta z_{2}}\left(Q_{3}-Q_{2}+\lambda_{2}\sqrt{H_{3}}\right)\\ \frac{-gA}{\Delta z_{3}}\left(H_{4}-H_{3}\right)-\frac{\tau }{2\phi A}Q_{3}\left|Q_{3}\right| \end{array}\right]$$

Notice that, due to mass conservation, $Q_{l_{1,2}}$ must satisfy the next relation:

(6) $$Q_{l_{1,2}}=Q_{b_{1,2}}-Q_{a_{1,2}}$$

where $Q_{b_{1,2}}$ and $Q_{a_{1,2}}$ are the flows in an infinitesimal length before and after the leak, respectively.

#### 2.1.3. Pipeline Equivalent Straight Length

It is worth pointing out that the mathematical model (5) assumes a straight pipe. This is not a loss of generality because even if the pipe is not straight, it is possible to obtain an equivalent straight length (*ESL*) of the pipe. The *ESL* is the straight length of a virtual pipe (with the same parameters as the original duct) that would give rise to the same pressure drop as the real pipeline. Such an equivalence is calculated by considering losses due to each “non-straight element” (i.e., fitting) in accordance with the Darcy–Weisbach formula [2,16,17]:

(7) $$l_{e}=\frac{\phi }{\tau }K$$

where, $l_{e}$ means the equivalent straight length of a specific fitting, *K* is the so-called loss coefficient parameter, which is normally provided by the pipe manufacturer, and τ is the friction coefficient. Thus, the total ESL of the pipe, $L_{e}$, can be calculated as follows:

(8) $$L_{e}=L_{r}+\frac{\phi }{\tau }\sum_{i=0}^{n_{f}}K_{i}$$

where $L_{r}$ stands for the sum of all pipeline straight length elements, $K_{i}$ is the fitting loss coefficient for the *i*-th fitting, and $n_{f}$ the number of the pipeline fittings.

#### 2.1.4. Friction Model

The friction factor (τ in Equation (1)) represents the loss of pressure of a fluid due to the interactions between the fluid and the internal surface roughness of the pipe. Thus, such a friction factor is a function of the Reynolds number, Re, and the pipe’s roughness, ϵ [18,19].

In many practical cases, τ is deemed to be a constant value, which is commonly taken from the Moody chart; nevertheless, in pipes with a relative roughness usually less than $1\times 10^{-3}$ m, the zone where the friction factor is almost constant (i.e., the complete turbulence zone), is difficult to reach. Consequently, when the *LDI* scheme is applied to a plastic pipe, it is preferable to obtain a more accurate friction value by using a formula or an algorithm to estimate such value. In this work, the authors propose the use of the well know Swamee–Jain equation to directly calculate the coefficient of friction:

(9) τi(Qi)=0.25log10ϵ3.7ϕ+5.74Rei0.92

where subscript *i* denotes the section number of the pipeline (see Figure 2). The Reynolds number is, in turn, function of the flow rate, $Q_{i}$, as follows:

(10) Rei=QiϕνA

where, ν is the kinematic viscosity of the water. Notice that, due to leak occurrence, the flow rate is different in each pipeline section (see Figure 2), causing a significant deviation of the friction factor value (since the working plastic pipe area is commonly in the transition zone). Therefore, it is important to introduce Equations (9) and (10) in the mathematical model to calculate, at any sampling time, the friction coefficient due to the flow variations in each *i*-th section.

### 2.2. Two Simultaneous Leak Problem Statement

In this work, the two simultaneous leak case is considered, i.e., a couple of leaks can appear in a pipeline at locations: $z_{1}\in \left(0,L\right)$ and $z_{2}\in \left(0,L\right)$, with $z_{2}>z_{1}$. Thus, the problem is reduced to the size estimation of the pipe sections: $\Delta z_{1}$ ($\Delta z_{1}=z_{1}$) and $\Delta z_{2}$ ($\Delta z_{2}=z_{2}-z_{1}$), see Figure 2.

Now, Equation (5) can be written in compact form as follows:

(11) $$\begin{array}{ccc} \dot{\zeta } & = & \xi (\zeta )+\rho (\zeta )\gamma \\ \Psi & = & \vartheta (\zeta ) \end{array}$$

where $\zeta ={\left[\zeta_{1}\;\zeta_{2}\;\zeta_{3}\;\zeta_{4}\;\zeta_{5}\right]}^{T}={\left[Q_{1}\;H_{2}\;Q_{2}\;H_{3}\;Q_{3}\right]}^{T}\in R^{5}$ is the state vector, $\gamma ={\left[H_{in}\;H_{out}\right]}^{T}\in R^{2}$ is the input vector, and $\Psi ={\left[Q_{in}\;Q_{out}\right]}^{T}\in R^{2}$ is the output vector for some functions ξ, ρ, and ϑ.

The leak diagnosis problem for two simultaneous leaks appearing in a pipeline can then amount to the estimation of parameters $\Delta z_{1}$, $\Delta z_{2}$, $\lambda_{1}$, and $\lambda_{2}$ in (5). Let us consider those parameters as new state variables with constant dynamics [11], that is: if $\theta ={\left[z_{1}\;z_{2}\;\lambda_{1}\;\lambda_{2}\right]}^{T}$ then $\dot{\theta }=0$. This results in an extended state: $x={\left[\zeta \;\theta \right]}^{T}={\left[Q_{1}\;H_{2}\;Q_{2}\;H_{3}\;Q_{3}\;\Delta z_{1}\;\Delta z_{2}\;\lambda_{1}\;\lambda_{2}\right]}^{T}=:{\left[x_{1}\;x_{2}\;x_{3}\;x_{4}\;x_{5}\;x_{6}\;x_{7}\;x_{8}\;x_{9}\right]}^{T}\in R^{9}$. Then, considering an unidirectional flow given by $Q_{i}\left|Q_{i}\right|=Q_{i}^{2}$, the extended state representation of (11) can take a form as follows:

(12) $$\begin{array}{ccc} \dot{x} & = & f(x,u)\\ y & = & h(x) \end{array}$$

where $u≐{\left[H_{1}\;H_{4}\right]}^{T}≐{\left[u_{1}\;u_{2}\right]}^{T}$, and *f*, *g* are differentiable vector fields with the following structure:

(13) $$f\left(x,u\right)=\left[\begin{array}{c} -\frac{gA}{x_{6}}\left(x_{2}-u_{1}\right)-\mu_{1}\left(x_{1}\right)x_{1}^{2}\\ -\frac{b^{2}}{Agx_{6}}\left(x_{3}-x_{1}+x_{7}\sqrt{x_{2}}\right)\\ -\frac{gA}{x_{8}}\left(x_{4}-x_{2}\right)-\mu_{2}\left(x_{3}\right)x_{3}^{2}\\ -\frac{b^{2}}{Agx_{8}}\left(x_{5}-x_{3}+x_{9}\sqrt{x_{4}}\right)\\ -\frac{gA}{L-x_{6}-x_{8}}\left(u_{2}-x_{4}\right)-\mu_{3}\left(x_{5}\right)x_{5}^{2}\\ O^{4\times 1} \end{array}\right]\;h\left(x\right)=\left[\begin{array}{c} y_{1}\left(x\right)\\ y_{2}\left(x\right) \end{array}\right]=\left[\begin{array}{c} Q_{1}\\ Q_{3} \end{array}\right]=\left[\begin{array}{c} x_{1}\\ x_{5} \end{array}\right]$$

where $O^{4\times 1}$ is the 4×1 zero matrix, $\mu_{i}=\frac{\tau_{i}}{2\phi A}$ is computed as in Equation (9), and $\tau_{1,2,3}$ are functions of $x_{1}$, $x_{3}$, and $x_{5}$, respectively.

## 3. State Vector Reconstruction Based on Input–Output Numerical Differentiation

### 3.1. Observability Discussion

For the one leak case, the so-called observability rank condition is satisfied, and such a property does not depend on the inputs [12]. However, in the case of two simultaneous leaks (or even more), this is no longer true, since the states are not distinguishable by applying constant inputs, and thus a persistent input is required [11].

In fact, to reconstruct the actual state vector *x* of (12), one can compute time derivatives of the output as functions of state variables as well as input and its time derivatives (using Equation (13)), such that an invertible map with respect to the full state vector *x* is obtained by applying an appropriate input. More precisely, by considering the two simultaneous leak case described by (12), we have two input variables and two output variables, from which input–output time derivative vectors can be generally defined up to orders *p*, $p^{\prime }$ for the input and *q*, $q^{\prime }$ for the output, as:

(14) $$U_{\left(p,p^{\prime }\right)}\left(t\right):={\left[u_{1}\;{\dot{u}}_{1}\;\cdots \;u_{1}^{\left(p\right)}\;u_{2}\;{\dot{u}}_{2}\;\cdots \;u_{2}^{\left(p^{\prime }\right)}\right]}^{T}$$

and

(15) $$Y_{\left(q,q^{\prime }\right)}\left(t\right):={\left[y_{1}\;{\dot{y}}_{1}\;\cdots \;y_{1}^{\left(q\right)}\;y_{2}\;{\dot{y}}_{2}\;\cdots \;y_{2}^{\left(q^{\prime }\right)}\right]}^{T}$$

Clearly, from (13), the output time derivatives depend on the state and input time derivatives, so that we can get:

(16) $$Y_{\left(q,q^{\prime }\right)}\left(t\right)=\Gamma \left(x,U_{\left(p,p^{\prime }\right)}\right)$$

for some *p*, $p^{\prime }$, given *q*, $q^{\prime }$.

On this basis, observability somehow means that this relationship is invertible and that it is possible to find elements among the components of Γ defining an invertible map with respect to *x* [20,21]. Specifically, if such an inverted map exists, and the input–output and the corresponding time derivatives are known, then, it is possible to compute each independent state in (16) as follows:

(17) $$x=\Gamma^{-1}\left(U_{\left(p,p^{\prime }\right)},Y_{\left(q,q^{\prime }\right)}\right)$$

Notice that, in general, it can be of interest to avoid or limit time derivatives of the input, but assuming that they are available or can be estimated in the same way as time derivatives of y is enough for our present application.

It should be noted that for this LDI problem, it is even enough to obtain an expression by only relating the input, output, and their time derivatives with leak parameters. We will propose this in Section 3.3, with a similar procedure as *elimination* (which, conversely to realization, consists of deriving an externally equivalent representation not containing the state [22]). We will even see how to recover the full state vector. However, let us first discuss the way to obtain input–output time derivatives.

### 3.2. Numerical Differentiation with Annihilators

The *LDI* scheme proposed in this work is based on Equation (17), that is, the injection of the inputs, outputs, and their time derivatives. Although there are multiple algorithms to numerically compute a time derivative, here we use a numerical differentiation with annihilators [23] due to its inherent noise rejection properties. Hereinafter, a brief explanation of such an algorithm is given, which is proposed for the first time in this paper, and it is described by Equation (25).

Let $\gamma^{m}\left(t\right)$ denote the *m*-th order derivative of a smooth signal γ(t) defined on an interval $I\subset R^{+}$. The signal γ(t) could represent a measurement variable corrupted by some noise, whose derivatives are not directly available.

Ignoring the noise for a moment, let γ(t) be an analytical function on I. So, without any loss of generality, it is possible to consider the truncated Taylor expansion at t=0:

(18) $$\gamma \left(t\right)=\sum_{i=0}^{m}a_{i}\frac{t^{i}}{i!}+O\left(t^{m}\right)$$

where $a_{i}={\left.\frac{d^{i}\gamma \left(t\right)}{dt^{i}}\right|}_{t=0}$. This implies that γ(t) can be approximated by the polynomial $p_{m}\left(t\right)=\sum_{i=0}^{m}a_{i}\frac{t^{i}}{i!}$.

In this way, the *i*-th order time derivative estimation of γ(t) can be tackled as a parameter estimation problem for $p_{m}\left(t\right)$. Using the method described in [23], it is possible to calculate each $a_{i}$ (i=0,⋯,m) independently, reducing in this manner the sensitivity to noise and numerical computation errors which often appear in simultaneous estimation methods. Moreover, the independent calculation allows the use of higher order polynomials without the calculation of all their coefficients. The algorithm is described as follows (a complete explanation is given in [23]):

- Let $p_{m}\left(t\right)$ be the *m*-th order polynomial approximation of γ(t),
  (19) $$p_{m}=\frac{a_{0}}{0!}+\frac{a_{1}}{1!}t+\frac{a_{2}}{2!}t^{2}+\cdots +\frac{a_{m}}{m!}t^{m}$$
- Transforming Equation (19) into Laplace domain yields:
  (20) $$P_{m}=\frac{a_{0}}{s}+\frac{a_{1}}{s^{2}}+\frac{a_{2}}{s^{3}}t^{2}+\cdots +\frac{a_{m}}{s^{m+1}}$$
- In order to calculate the *i*-th time derivative approximation, $a_{i}$, it is necessary to first annihilate every $a_{x}$ (x>i) in (20), using the next operator:
  (21) $$\left[\prod_{l=0}^{m-i}\frac{d}{ds}\right]\cdot s^{m+1}$$
- Then, to annihilate every $a_{x}$ (x<i), the following operator is subsequently applied to Equation (20):
  (22) $$\left[\prod_{h=0}^{i}\frac{d}{ds}\right]\cdot s^{-1}$$
- Finally, the resulting equation (after applying Equations (20)–(22)) is:
  (23) $$\begin{array}{c} l{\left(-1\right)}^{i}\left(m-i\right)!i!a_{i}s^{-(i+1)}=\\ \sum_{h=0}^{i}\sum_{l=0}^{m-1+h}{\left(-1\right)}^{i-h}\left(\begin{array}{c} i\\ h \end{array}\right)\left(\begin{array}{c} m-i+h\\ l \end{array}\right)\frac{(i-h)!(m+1)!}{(i+1-h+l)!}s^{l}\frac{d^{l}P_{m}\left(s\right)}{ds^{l}} \end{array}$$
- Now, multiplying both sides of the above equation by $s^{-(m+1)}$ yields a polynomial taking the following form:
  (24) $$\frac{c_{m}}{s^{i+m+2}}a_{i}=\frac{1}{s}\frac{d^{m}P_{m}\left(s\right)}{ds^{m}}+\frac{c_{m-1}}{s^{2}}\frac{d^{m-1}P_{m}\left(s\right)}{ds^{m-1}}+\ldots +\frac{c_{0}}{s^{m+1}}P_{m}\left(s\right)$$
- Using the Cauchy rule for iterated integrals, the time domain expression for $a_{i}$ in Equation (24) yields:
  (25) $$a_{i}=\frac{(m+i+1)!}{c_{m}T^{m+i+1}}\int_{0}^{T}\left[t^{m}+\frac{c_{m-1}}{1!}\left(T-t\right)t^{m-1}+\ldots +\frac{c_{0}}{m!}{\left(T-t\right)}^{m}\right]p\left(t\right)\cdot dt$$

It should be noted that each constant $c_{j}$, j={0,1,…,m}, is obtained from Equation (23), and *T* represents a moving window of length *T* for the integrals. A short time window is sufficient to obtain accurate estimations. In addition, the iterated integrals work as low pass filters that provide a smoother form of highly fluctuating noises. Therefore, no previous knowledge on the statistical properties of the noise is required to filter it out.

### 3.3. Extended State Vector Reconstruction

The purpose of this section is to provide a synthesis of the proposed *LDI* scheme. In this sense, the method is based on the study of the structure of the input–output differential equation; thus, the problem is solved by exploiting the observability property of the system (13). First, a set of equations describing each state just as a function of the inputs, outputs, and the corresponding time derivatives is derived. Then, taking advantage of the filtering characteristic of the numerical differentiation exposed in Section 3.2, the input–output time derivatives are computed. Thus, the leak positions and magnitudes can be calculated by a pair of algebraic equations. In the next step, the algorithm used to derive this set of equations is described.

It is easy to check that the observability rank condition for the system (12) is satisfied (for the finite number of input–output time derivatives), only by applying a persistent input. More precisely, it is also easy to see that for an input of the form $A_{p}\sin\left(\omega t\right)$. This wave form is easy to achieve by a variable frequency drive, a commonly installed device in a pumping station.

The rank condition of (12) is fulfilled with p=3, $p^{\prime }=2$, q=4, and $q^{\prime }=3$ for Equations (14) and (15). Thus, the output derivatives as well as the inverse mapping can be computed as follows (perhaps the major difficulty of the algorithm is to obtain the inverse mapping due to the complexity of the resulting equation):

First, by construction of Equation (13), the state variables $x_{1}$ and $x_{5}$ are taken directly from the measurements:

(26) $$x_{1}=y_{1}$$

(27) $$x_{5}=y_{2}$$

Now, the following step is to take the time derivatives of Equations (26) and (27), by using (13) and replacing $x_{1}$, $x_{5}$ by outputs according to (26) and (27) solving the resulting equation for $x_{2}$ and $x_{4}$, respectively, yields expressions without $x_{1}$ and $x_{5}$:

(28) $$x_{2}=\Phi_{1}\left(x_{6},u_{1},y_{1},{\dot{y}}_{1}\right)$$

(29) $$x_{4}=\Phi_{2}\left(x_{6},x_{8},u_{2},y_{2},{\dot{y}}_{2}\right)$$

In the same way, the third step is to compute the derivative of Equation (28), substitute $x_{1}$, $x_{5}$, $x_{2}$, and $x_{4}$ according to (26), (27), (28), and (29), respectively. Solving this equation for $x_{3}$, yields an expression without $x_{1}$, $x_{5}$, $x_{2}$, $x_{4}$:

(30) $$x_{3}=\Phi_{3}\left(x_{6},x_{7},u_{1},{\dot{u}}_{1},y_{1},\ldots ,y_{1}^{\left(2\right)}\right)$$

Following the same steps, now for (29) and (30), it is possible to find an expression for $x_{7}$ and $x_{9}$ free of $x_{1}$, $x_{2}$, $x_{3}$, $x_{4}$, and $x_{5}$:

(31) $$x_{7}=\Phi_{4}\left(x_{6},x_{8},u_{1},\ldots ,u_{1}^{\left(2\right)},u_{2},y_{1},\ldots ,y_{1}^{\left(3\right)},y_{2},{\dot{y}}_{2},\right)$$

(32) $$x_{9}=\Phi_{5}\left(x_{6},x_{8},u_{1},\ldots ,u_{1}^{\left(2\right)},u_{2},{\dot{u}}_{2},y_{1},\ldots ,y_{1}^{\left(3\right)},y_{2},\ldots ,y_{2}^{\left(2\right)}\right)$$

The final step is to apply the same methodology for Equations (31) and (32), but now for solving for the states $x_{6}$ and $x_{8}$. In this way, it is feasible to obtain an expression of $x_{6}$ and $x_{8}$ just as a function depending on the input and output, and their time derivatives:

(33) $$x_{6}=\Phi_{6}\left(u_{1},\ldots ,u_{1}^{\left(3\right)},u_{2},{\dot{u}}_{2},y_{1},\ldots ,y_{1}^{\left(4\right)},y_{2},\ldots ,y_{2}^{\left(3\right)}\right)$$

(34) $$x_{8}=\Phi_{7}\left(u_{1},\ldots ,u_{1}^{\left(3\right)},u_{2},..,u_{2}^{\left(2\right)},y_{1},\ldots ,y_{1}^{\left(4\right)},y_{2}\ldots ,y_{2}^{\left(3\right)}\right)$$

At this point, we are able to recover the whole state with the acknowledgement of the input and output time derivatives calculated through Equation (25); this is achieved by first obtaining $x_{6}$ and $x_{8}$ (leak positions) from Equations (33) and (34). Once $x_{6}$ and $x_{8}$ are obtained, leak magnitudes, $x_{7}$ and $x_{9}$, can be computed by using (31) and (32). The rest of the state can then be recovered going backwards through Equations (30)–(28).

## 4. Experimental Results

In this section, experimental results are presented to evaluate the proposed *LDI* methodology. The experiments are performed using several databases from the pilot plant located at Cinvestav Guadalajara. A couple of different two simultaneous leak scenarios were emulated by opening different electrovalves located along the pilot plant. A general description of the pipeline prototype is presented below with a detailed description of each experiment.

### 4.1. Pilot Pipeline Description

**Figure 3:**
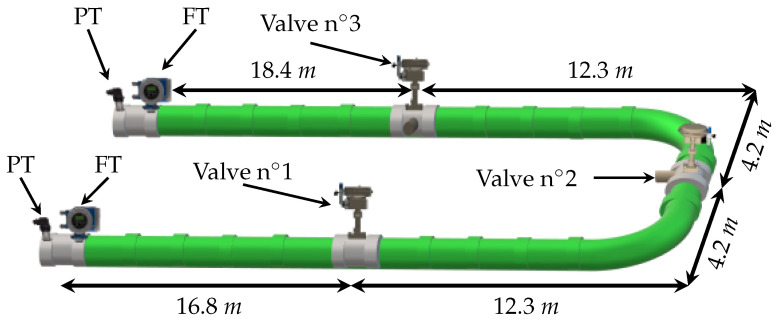
Schematic diagram of the pipeline’s prototype.

The layout of the pilot pressurized water pipe of Cinvestav Guadalajara, which is 68.2 [m] long (between sensors) with an internal diameter of $6.271\times 10^{-2}$ [m], thickness $1.27\times 10^{-2}$ [m], friction coefficient $1.66\times 10^{-2}$, pressure wave speed 358 [m/s], and gravity acceleration 9.81 [m/s${}^{2}$], is shown in Figure 3. The line is instrumented with two water-flow (FT) and pressure-head (PT) sensors at the inlet and outlet of the pipe. To emulate the leak, three control valves at position 16.8, 33.3, and 49.8 [m] are installed together with a electronic-based actuator to practically set any opening of the valve.

The prototype is manufactured with a plastic material known as polypropylene copolymer random, for which technical characteristics can be found in [24]. It is integrated with a store tank of $7.5\times 10^{-1}$ [m${}^{3}$], a hydraulic pump of 5 HP, and a variable-frequency driver (*VFD*) which controls the pressure in the system through the rotational speed of the pump motor (more details about the pipeline prototype can be found in [25]).

To ensure the application of a persistent input, experiments with operation point variations are carried out by the pump variation via the *VFD* (specifically, a change in the form of $A_{p}\sin\left(\omega t\right)$, where ω stands for the angular frequency induced via the *VFD*). Table 1 summarizes the pipeline’s main parameters.

### 4.2. LDI Results

Hereinafter, two off-line examples of the *LDI* scheme are displayed. Both results were carried out by taking data from the pipeline prototype previously described. The experiment was performed as follows: Pump 1 is started in a steady state operation during the first 65 [s] approximately. After that, it begins to operate in some unsteady state, namely a sine-like pressure signal is introduced, just exactly like a persistent input in the sense of [11]. This sine signal was experimentally obtained by setting up the pump controller as follows:

(35) $$\begin{array}{ccc} VFD(t) & = & 60[Hz],\forall t\leq 65[s]\\ VFD(t) & = & 50+5sin(2.7313t)[Hz],\forall t>65[s] \end{array}$$

At t≈60 [s], two leaks were induced simultaneously at the opening of the control valves in the pilot plant.

The leak position estimations were undertaken by the injection of the inputs and outputs for which a low pass filter was previously applied ($u_{1}$, $u_{2}$, $y_{1}$, and $y_{2}$ in (13)), and the corresponding derivatives together with Equations (33) and (34), respectively.

Then, the leak magnitudes were also estimated by using Equations (31) and (32). It is possible to recover the whole state going backwards using Equations (30)–(28). As stated earlier, the time derivatives were computed using the methodology discussed in Section 3.2.

#### 4.2.1. Experiment 1: Leaks Induced in Valves $n^{\circ }1$ and $n^{\circ }3$

**Figure 4:**
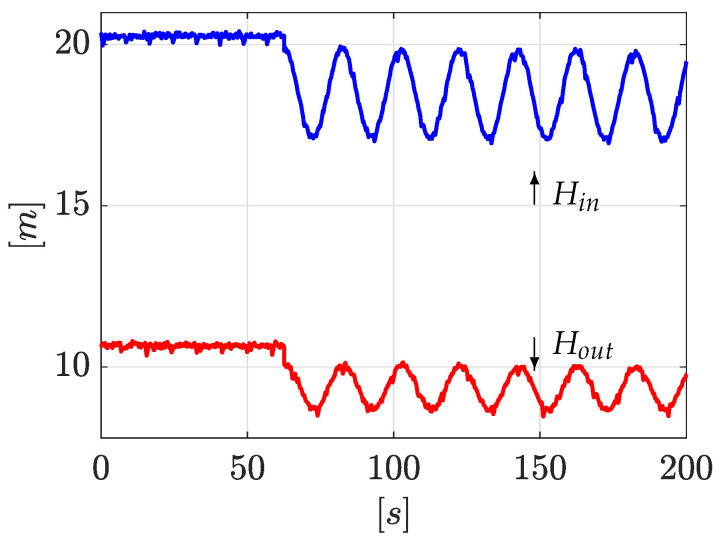
Pressure heads $H_{in}$ and $H_{out}$.

The first experiment consists of simultaneously inducing two leaks in valve $n^{\circ }1$ and valve $n^{\circ }3$ (see Figure 3). The algorithm starts once the leak is detected. This initial detection is obtained when a deviation between the upstream and downstream flow is detected $|Q_{in}-Q_{out}|>\delta$, where δ is a constant threshold defined by the designer (normally related to the signal-noise ratio): here $\delta =1\times 10^{-4}$ [m${}^{3}$/s]. Immediately afterwards, a sinusoidal wave form as (35) is applied to ensure enough observability of the model (13) via persistent inputs (see Section 3.1). Once the sinusoidal steady state has been reached, the *LDI* scheme starts. Figure 4 shows the time evolution of the pressure head at the inlet and outlet of the pipe (system input, $H_{in}=u_{1}$ and $H_{out}=u_{2}$). As it can be seen, the *LDI* algorithm begins close to 65 [s].

**Figure 5:**
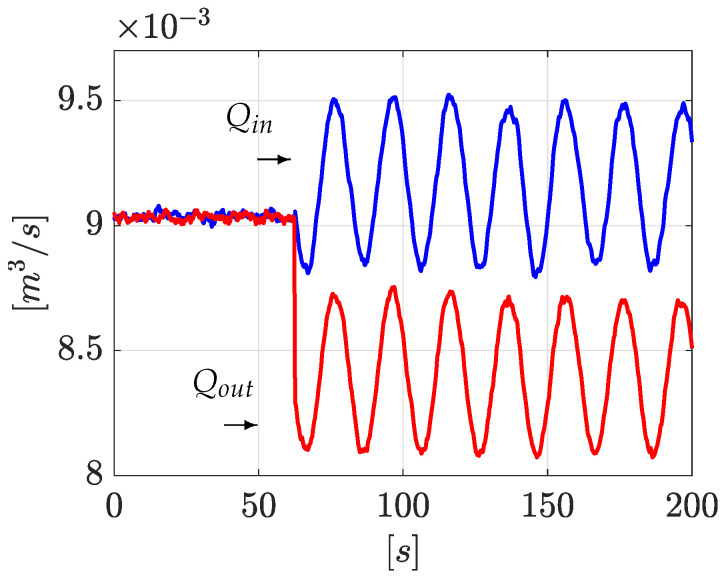
Flow rates $Q_{in}$ and $Q_{out}$.

**Figure 6:**
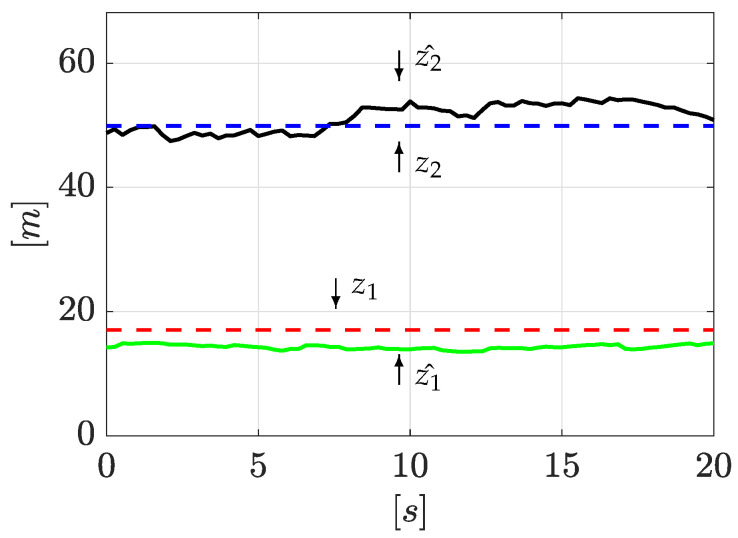
Estimation of leak positions.

Following with the same idea, Figure 5 shows the inlet and outlet flows (system output $Q_{in}=y_{1}$ and $Q_{out}=y_{2}$). It is clear that, due to the physical nature of the leak phenomena, the inlet flow is separated from the outlet flow:

Now, once the input and output time derivatives have been computed with Equations (25), (33), and (34), then they are used to reconstruct the leak positions. For clarity, just one signal cycle ($T_{in}=20$ s), as seen in Figure 6, is enough to correctly locate the two leaks, despite signal noise. To quantify the accuracy of the leak position estimation, a Mean Absolute Error index is applied. For the first leak, the estimation accuracy is 96.62% (with respect to the whole pipeline length), whereas the second leak localization accuracy is 96.33%. As Figure 6 shows, one cycle has demonstrated to be enough to isolate the leak well despite signal noise.

**Figure 7:**
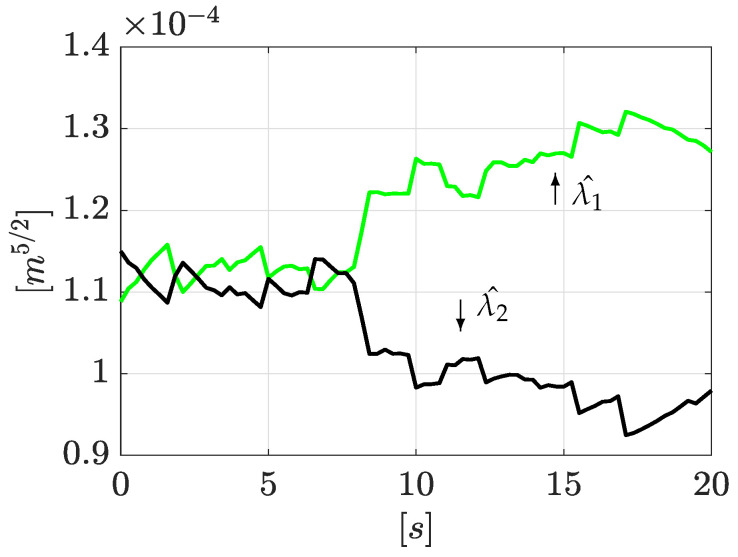
Estimation of leak magnitudes.

Similarly, once the leak positions are obtained, it is possible to reconstruct the leak magnitudes using Equations (31) and (32). The corresponding results are shown in Figure 7, where estimated magnitudes are set around $1.3\times 10^{-4}$ [m${}^{5/2}$/s] and $0.95\times 10^{-4}$ [m${}^{5/2}$/s], respectively. The outflow computed by using Equation (4) for each leak is consistent with the total outflow.

Notice that for the *LDI* problem, the estimation of the leak parameters of both leaks is enough, but, as mentioned before, it is also possible to recover the remaining states of (13), going backwards from Equations (30) and (28).

#### 4.2.2. Experiment 2: Leaks Induced in Valve $n^{\circ }1$ and $n^{\circ }2$

**Figure 8:**
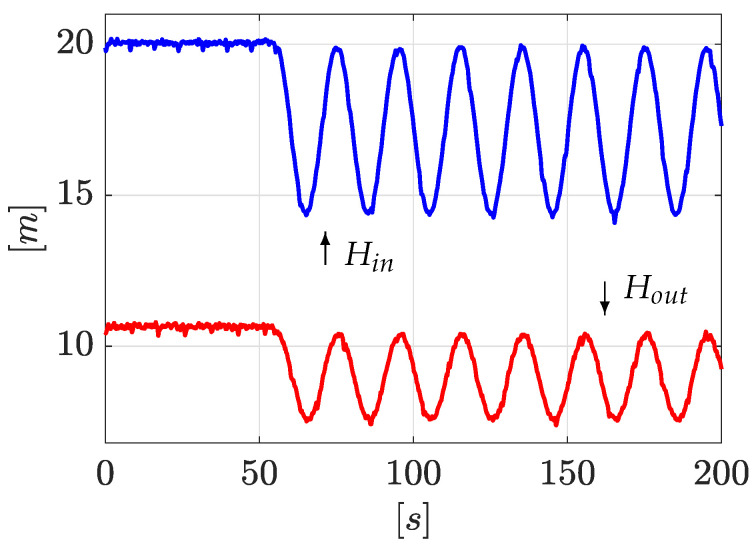
Pressure heads $H_{in}$ and $H_{out}$ (2nd experiment).

To better illustrate the effectiveness of the method, a second experiment is exposed. The experiment setup was exactly the same as in Section 4.2.1 except that two leaks are now induced in valve $n^{\circ }1$ and valve $n^{\circ }2$ (see Figure 3). Figure 8 shows the time evolution of the pressure head at upstream and downstream, respectively. In the same way as before, the *LDI* algorithm starts when a flow deviation exceeds a predefined threshold, δ, ($|Q_{in}-Q_{out}|>\delta$).

**Figure 9:**
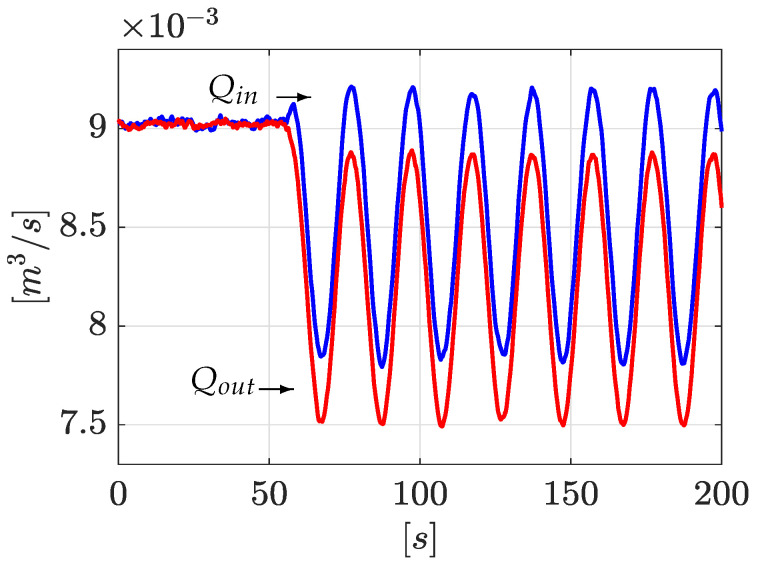
Flow rate $Q_{in}$ and $Q_{out}$ (2nd experiment).

Figure 9 shows the corresponding flow rate evolution at upstream and downstream. As stated above, due to the physical nature of the leak phenomena, the inlet flow is separated from the outlet flow.

**Figure 10:**
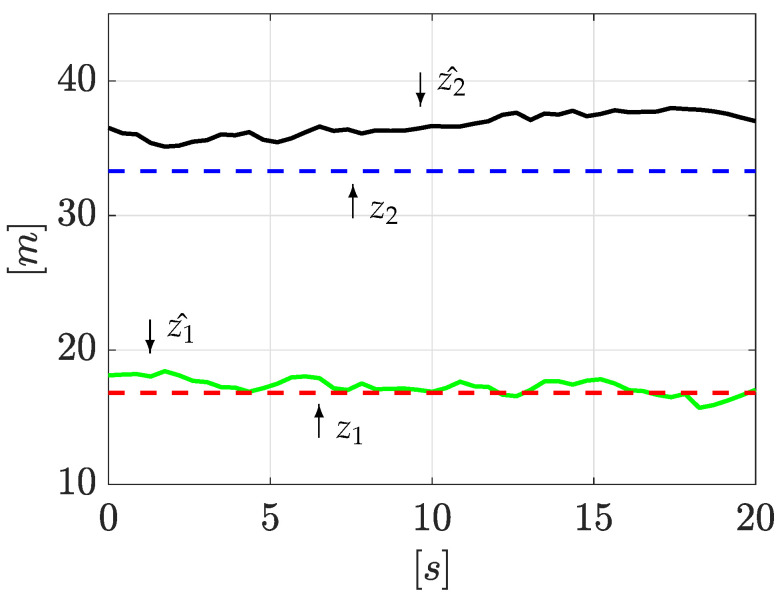
Estimation of leak positions (2nd experiment).

As performed above, once the algorithm starts, the input and output time derivatives are computed following the algorithm explained in Section 3.2. Subsequently, Equations (33) and (34) are used to obtain the leak positions. The results are presented in Figure 10. As before, the leak positions are well estimated despite signal noise in just one signal cycle ($T_{in}=20$ s). Here, the MAE yields an estimation accuracy for the first leak of 95.01%, while the second leak position presents an estimation accuracy of 97.94%.

**Figure 11:**
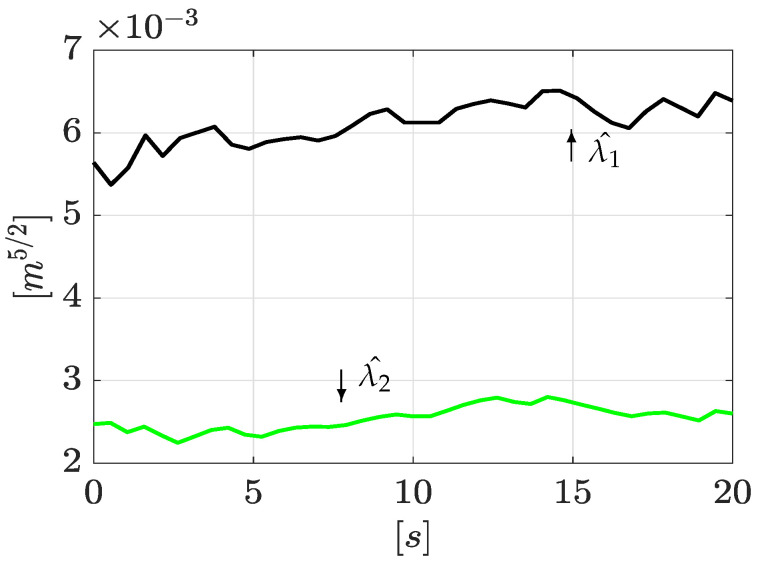
Estimation of leak magnitudes (2nd experiment).

Continuing with the algorithm, the leak magnitudes are computed with Equations (31) and (32). These results are shown in Figure 11.

## 5. Conclusions

The simultaneous leak detection and isolation problem is currently an open and challenging problem. Even though extensive research in the field is currently in progress, to the best of our knowledge, only simulation results have been reported until now. One of the reasons is that the distinguishability of two (or more) simultaneous leaks depends on the input.

In this work, an *LDI* methodology for the two simultaneous leak detection and isolation has been proposed based on an algebraic observer that uses the injection, the inputs and outputs of the system, and the corresponding time derivatives, by applying an appropriate input. The time derivatives are computed using *Numerical Differentiation with Annihilators* and the approach has been successfully applied to real data. This methodology could be extended to more general cases of simultaneous leaks in real operational conditions, such as the case of the *SIAPA* aqueduct in Guadalajara, Mexico.

## Figures and Tables

**Table 1:** Pipeline’s main parameters.

Parameter	Symbol	Value	Units
Pipeline length	$L_{r}$	68.2	[m]
Upstream to valve $n^{\circ }1$	$z_{1}$	16.8	[m]
Upstream to valve $n^{\circ }2$	$z_{2}$	33.3	[m]
Upstream to valve $n^{\circ }3$	$z_{3}$	49.8	[m]
Internal diameter	ϕ	$6.271\times 10^{-2}$	[m]
Pipe roughness	ϵ	$7\times 10^{-6}$	[m]
Friction factor	τ	$1.66\times 10^{-2}$	[dimensionless]
Pressure wave speed	*b*	358	[m/s]
Gravity acceleration	*g*	9.8	[m/s${}^{2}$]
